# Maternal hyperglycemia disturbs neocortical neurogenesis via epigenetic regulation in C57BL/6J mice

**DOI:** 10.1038/s41419-019-1438-z

**Published:** 2019-03-01

**Authors:** Shufang Ji, Wenjuan Zhou, Xian Li, Shangming Liu, Fuwu Wang, Xinyue Li, Tiantian Zhao, Guangyu Ji, Jingyi Du, Aijun Hao

**Affiliations:** 1 Key Laboratory of the Ministry of Education for Experimental Teratology, Shandong Provincial Key Laboratory of Mental Disorders, Department of Human Anatomy and Histoembryology, School of Basic Medical Sciences, Shandong University, Jinan, Shandong China; 2 Foot and Ankle Surgery Center of Shandong University and Department of Hand and Foot Surgery, The Second Hospital of Shandong University, Jinan, Shandong China

## Abstract

Offspring of mothers with hyperglycemia during pregnancy have a higher incidence of long-term neuropsychiatric disorders than offspring from a normal pregnancy, indicating that neocortical neurogenesis might be affected by maternal hyperglycemia. A paucity of study evaluating the effects of hyperglycemia on neocortical neurogenetic differentiation of neural stem cells, and the mechanism remains unclear. We sought to investigate the the roles and possible molecular mechanism of maternal hyperglycemia on neocortical neurogenetic differentiation of neural stem cells. We established a mouse model of a hyperglycemic pregnancy to study effects of intrauterine exposure to maternal hyperglycemia on neocortical neurogenesis. We observed morphological changes in the neocortex and detected the neurogenetic differentiation of neural stem cells in offspring affected by high glucose levels. We investigated the regulatory network between epigenetic modification and transcription factors in differentiated neural stem cells under hyperglycemic conditions. Maternal hyperglycemia disturbs neocortical lamination in some non-malformed offspring. Our results suggested that hyperglycemia altered the early-born neuron fate and the distribution of newborn neurons in deep layers by promoting the earlier differentiation of neural stem cells. Altered histone acetylation and its regulation on the transcription of proneural genes might be correlated to the disrupted differentiation of neural stem cells and altered distribution of newborn projection neurons in the neocortex. Our data raised the possibility that maternal hyperglycemia in pregnancy disturbs the laminar distribution of neocortical projection neurons in some non-malformed offspring via epigenetic regulation on neural stem cell differentiation and the birthdate of neocortical neurons.

## Introduction

Hyperglycemia in pregnancy (HIP) has been demonstrated to increases risk of neurodevelopmental disorders in the offspring^1–3^. The severity of harmful effects on the neurodevelopment depends on the degree of hyperglycemia^4–6^. During development, the embryonic nervous system is susceptible to elevated blood glucose levels. Neurulation and corticogenesis are two critical stages during the development of the central nervous system^7,8^. Severe hyperglycemia in early pregnancy affects the closure of the embryonic neural tube and subsequently increases the risk of neural tube defects (NTDs)^9–11^. Moderate hyperglycemia does not substantially increase the risk of NTDs, and more offspring with a relatively normal phenotype are produced^12^. Considering that offspring from hyperglycemic pregnancy have higher incidences of long-term neuropsychological disorders and cognitive dysfunction than the offspring from a normal pregnancy^13,14^, we speculated that the process of neocortical development might be affected by hyperglycemia in some offspring with a normal phenotype from mothers with moderate hyperglycemia during pregnancy. Our collaborators and we previously focused on the effects of maternal hyperglycemia on embryonic neurodevelopment, primarily the effects of severe hyperglycemia on the incidence of NTDs and the mechanism by which high glucose levels cause NTDs^15–23^. However, few studies have examined the effects of moderate hyperglycemia on neurodevelopment in offspring without obvious NTDs. The incidence of gestational diabetes continues to increase, which has an enormous influence on embryonic neurodevelopment^24^. Fortunately, the incidence of NTDs has decreased to a certain extent, which has benefitted from modified glucose control and the widespread use of folic acid^25^. However, the long-term neuropsychiatric disorders in the non-malformed offspring affected by maternal hyperglycemia require further study. In this paper, our intent is to discover the mechanism of embryonic neurodevelopment disorders in non-malformed offspring from mothers with hyperglycemia in pregnancy.

The normal laminar structure of the cerebral cortex and the formation of complex neuronal circuits are essential for advanced central nervous system functions^26,27^. Normal development of the embryonic cerebral cortex plays a decisive role in the future physiological function of the central nervous system^28,29^. The dorsal pallium of the telencephalon gives rise to the neocortex^30^. Neocortical neurogenesis is a well-ordered process of the proliferation and differentiation of neural stem cells (NSCs) from the ventricular zone (VZ) and migration of newborn neurons outward^31^. Early-born neurons start to migrate radially in an orderly manner at embryonic day 12.5 (E12.5) in mice, forming the deep cortex (layer 6 first, then layer 5). Later born neurons begin to move to the superficial layers (layers 2–4) at E14.5^32^. The entire migration process is completed after birth. According to previous studies by Yuan et al. from our group, hyperglycemia in pregnancy increases the expression of the neuronal maker Tuj1 in the E13.5 dorsal telencephalon^21^. However, no relevant studies have examined how high glucose affects the neurogenetic differentiation and the subsequent birth and laminar distribution of neocortical neurons.

Epigenetic regulation plays an important role in the development of neocortex, including histone modification, DNA methylation, and non-coding RNAs^33–37^. Several studies on NTDs induced by maternal hyperglycemia have identified variations in epigenetic modifications^13^, particularly changes in histone acetylation. Our collaborators, Dheen et al., observed decreased acetylation of histone 3 on lysine 9 in proliferating NSCs exposed to severe hyperglycemia^38^. Yang et al. reported increased acetylation of histone 3 at several sites in embryos from diabetic mice^39^. However, the influence of moderately high glucose levels on the epigenetic mechanism controlling NSC differentiation during the development of the neocortical layers has not been identified. Among the epigenetic modifications that affect the expression of transcription factors (TFs), histone modifications are particularly complicated. Complex and dynamic regulatory networks between histone modifications and TFs regulate the proliferation and differentiation of NSCs during neocortical neurogenesis^33^. The orderly transcription of proneural genes guides the differentiation of new neurons in the neocortex^40–45^. Histone acetylation plays an important role in the transcription activation of proneural bHLH genes during the differentiation of NSCs into neurons^46^. In the present study, we detected changes in histone acetylation and their regulatory effects on the transcription of proneural genes under hyperglycemic conditions.

Adverse effects of hyperglycemia on embryonic neurodevelopment have far-reaching influences on the health of the offspring, which poses a serious threat to public health. Glycemic control during pregnancy does not completely avoid the risk of neurodevelopment disorders in offspring. Our study investigated the mechanism underlying the anomalous neocortical neurogenesis in non-malformed offspring of mothers with hyperglycemia in pregnancy. For the first time, moderate hyperglycemia was shown to disturb the laminar distribution of newborn neurons in the neocortex. Moreover, high glucose influenced the differentiation of NSCs, leading to changes in the birthdate and laminar distribution of neocortical neurons. We explored the mechanism regulating histone modification during the differentiation of NSCs affected by hyperglycemia. These findings improve our understanding of the relationship and network between epigenetic modifications and the transcription of TFs during the differentiation of NSCs under hyperglycemic conditions. We hope to provide targets for the diagnosis, intervention and reversal of embryonic neural dysplasia caused by hyperglycemia. We also describe methods for studying the influence of other maternal and environmental factors on the development of offspring, and provide possible candidate markers for the prevention and treatment of adverse outcomes.

## Results

### The phenotypes of the fetuses and the incidence of NTDs in the HIP group

In the present study, moderate hyperglycemic female C57BL/6J mice were mated with normal males, and the phenotypes of the fetuses were investigated. At E11.5, the fasting blood glucose level (FBG) of the HIP group (9.307 ± 0.796 mmol/l) was higher than the control group (5.719 ± 0.834 mmol/l) (Table 1). The incidence of NTDs (Table 1) in the HIP group was 8.97%, which was higher than the control group but less than the severe diabetes group (with fasting blood glucose levels exceeding 16.7 mmol/l) in our previous experiments^21^. Exposure to maternal hyperglycemia is confirmed as an independent risk factor for long-term neuropsychiatric morbidity in the offspring^4^. Thus, it is considerable to study the effects of maternal hyperglycemia on aberrant neurodevelopment. So we further detected the effects of maternal hyperglycemia on embryonic corticogenesis.

**Table 1:** Maternal blood glucose level and fetuses phenotype at E11.5

Groups	Litters number	Maternal fasting blood glucose level (mmol/l)	Implantations number	Alive fetuses		NTDs	
				number	ratio (%)	number	ratio (%)
Control	16	5.719 ± 0.834	123	118	95.9	2	1.69
HIP	15	9.307 ± 0.796	117	91	77.7	8	8.79

Maternal FBG levels are reported as the mean ± SD; *p* < 0.0001. Numbers of implantations number, alive fetuses and NTDs are reported as absolute numbers. The ratio of alive fetuses = number of alive fetuses/number of implantations × 100%. The ratio of NTDs = number of NTDs/number of alive fetuses × 100%

### Maternal high glucose disturbs neocortical lamination in non-malformed offspring

**Fig. 1:**
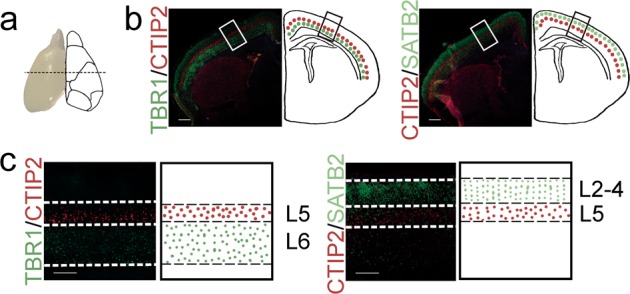
Analysis of the laminar structure of the cerebral cortex. **a** Coronal sections of the cerebrum were collected from the dotted line approximately indicated rostrocaudal axial position. **b** The laminar structure of the cerebral cortex was examined by immunofluorescence staining for neocortical identity makers. Scale bars, 400 μm. **c** Images of enlarge corresponding area of the neocortical laminar structure from the rectangular box indicated position of **b**. Scale bars, 100 μm

**Fig. 2:**
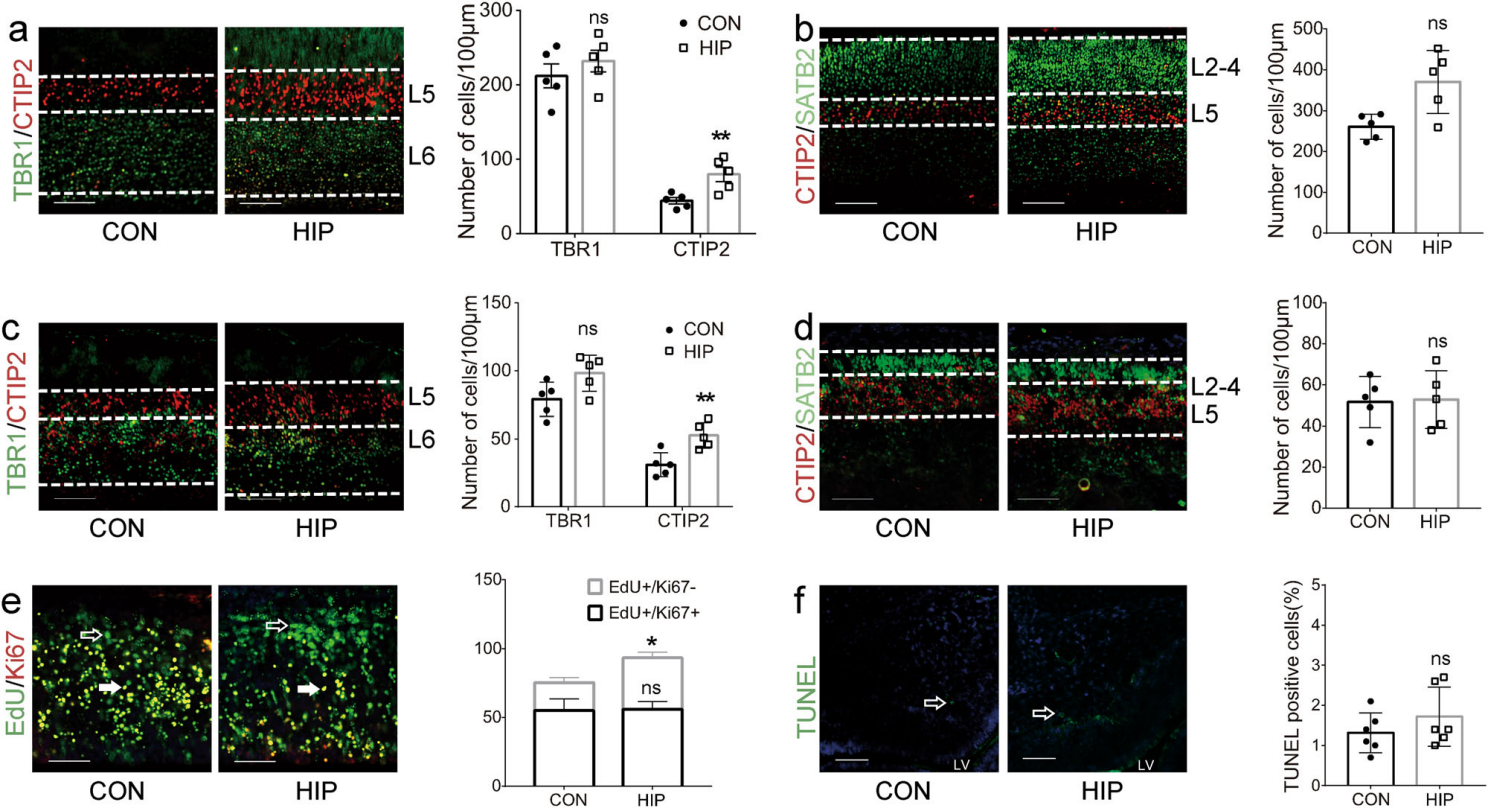
Effects of maternal hyperglycemia on the laminar distribution of neocortical neurons. **a** Numbers of TBR1 + and CTIP2 + cells in the cerebral cortex of neonatal (P0) HIP mice compared with the control group (*p* = 0.4286 and *p* = 0.0050, respectively). **b** Numbers of SATB2 + cells in the cerebral cortex of neonatal (P0) HIP mice compared with the control group (*p* = 0.0705). **c** Numbers of TBR1 + and CTIP2 + cells in the cerebral cortex of E17.5 HIP mice compared with the control group (*p* = 0.1109 and *p* = 0.0035, respectively). **d** Numbers of SATB2 + cells in the cerebral cortex of E17.5 HIP mice compared with the control group (*p* = 0.8631). **e** The percentages of proliferating and quiescent (exit cell cycle) cells were analyzed by staining NSCs with EdU and Ki67 in the E13.5 dorsal telencephalon (*p* = 0.6667 and *p* = 0.0382, respectively). **f** The apoptotic cells staining in VZ and SVZ of the E13.5 dorsal telencephalon were stained with TUNEL and counted (*p* = 0.3739). Data represent the mean of five independent experiments ± SD. *p < 0.05 vs. control; **p < 0.01 vs. control; ns, no significant difference vs. control. Scale bars, 100 μm

We detected the laminar structure of the neocortex in non-malformed fetal and neonatal offspring of moderate hyperglycemic pregnant mice. As shown in Fig. 1a, coronal sections of the telencephalon were acquired from postnatal 1 day (P1) mice from the dotted line approximately indicated rostrocaudal axial position^47^. We observed the laminar structures of the cortex by performing immunofluorescence staining with a cortical laminar marker, as shown in Fig. 1b. The enlaged image of laminar structure of the neocortex from the rectangular box indicated position of Fig. 1b is shown in Fig. 1c. The neocortical projection neurons in the deep layers, layer 6 and layer 5, are TBR1-positive and CTIP2-positive, respectively. The neocortical projection neurons in the upper layers, layers 2–4, are SATB2-positive^33^. The number of early-born CTIP2-positive neocortical neurons significantly increased in P1 mice in the hyperglycemic group compared with the control group (Fig. 2a), while number of early-born TBR1-positive neurons and late-born SATB2-positive neurons was not significantly different from the control group (Fig. 2a, b). We further detected the layered structure of the neocortex at E17.5 to determine whether similar changes were observed during fetal neurogenesis. The number of early-born CTIP2-positive neocortical neurons was significantly increased in the hyperglycemic group compared with the control group, while number of early-born TBR1-positive neurons and late-born SATB2-positive neurons was not significantly different from the control group (Fig. 2c, d). Otherwise, we observed that the thickness of the layer 5 also changed significantly in P1 and E17.5 mice in the hyperglycemic group compared with the control group (Figure S1).

### Maternal high glucose alters the fate of early-born deep layer neurons by promoting NSC early exit from the cell cycle

We detected the proliferation and apoptosis of NSCs in the VZ and SVZ to further reveal how maternal high glucose levels alter the fate commitment of early-born deep layer neurons. As a pyrimidine analog, EdU is incorporated in place of thymidine in the newly synthesized DNA of dividing cells during S phase in mitosis. Ki67 is expressed in all actively proliferating cells throughout the cell cycle, except for G0 phase^48^. The ratio of EdU+/Ki67− cells among total EdU-positive cells increased in the offspring from a moderately hyperglycemic pregnancy, particularly in the SVZ (Fig. 2e). Simultaneously, the percentage of apoptotic cells was not significantly altered, as evidenced by the TUNEL analysis (Fig. 2f). Based on our results, moderate hyperglycemia might disturb the cell cycle and proliferating and quiescent populations of NSCs during neocortical neurogenesis.

### High glucose levels promote early exit from the cell cycle and the premature differentiation of NSCs in vitro

**Fig. 3:**
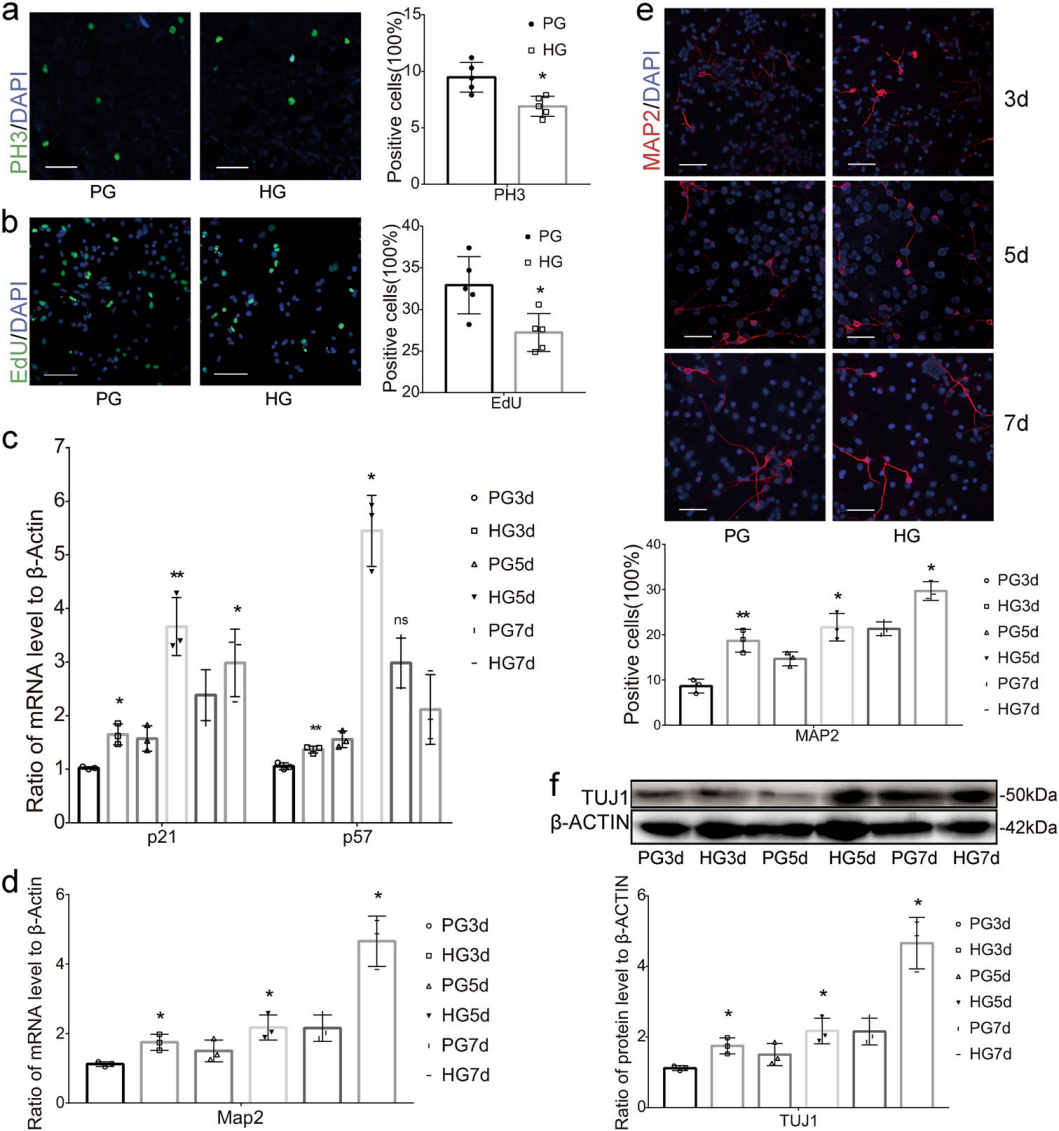
Effects of high glucose concentrations on the cell cycle exit and differentiation of NSCs. **a** The number of PH3+ cells in differentiated NSCs after 3 days of differentiation. **b** The number of EdU+ cells in differentiated NSCs after 3 days of differentiation. **c** Expression of the mRNAs encoding the cyclin-dependent kinase inhibitors (CKIs). The timescale indicates days after NSC differentiation. **d** Levels of the Map2 mRNAs in differentiated NSCs after 3, 5, and 7 days of differentiation. **e** Numbers of MAP2+ cells among differentiated NSCs after 3 and 5 days of differentiation. **f** Levels of the TUJ1 proteins in differentiated NSCs after 3, 5, and 7 days of differentiation. Data represent the mean of three independent experiments ± SD. *p < 0.05 vs. PG; **p < 0.01 vs. PG; ns, no significant difference vs. control. Scale bars, 50 μm

The determination of NSC fates during neocortical neurogenesis is coordinated with the mechanism regulating cell cycle exit. The high glucose treatment influenced the determination of NSC fates in vitro, similar to the altered embryonic neurogenesis process observed in offspring from a HIP. We counted the number of cells in the mitotic phase by staining cells for phosphohistone-H3 (PH3, an M-phase marker) and EdU to determine the effect of the high glucose treatment on the mechanism regulating the cell cycle exit of NSCs. The number of PH3-positive cells was decreased in high glucose-treated differentiated NSCs (Fig. 3a), meanwhile, the number of EdU-positive cells was reduced in high glucose-treated differentiated NSCs (Fig. 3b). We evaluated the expression of cyclin-dependent kinase inhibitors (CKIs) to verify the results. The high glucose treatment significantly increased the levels of CKI, p21, and p57 mRNAs (Fig. 3c). Consistent with the early exit from the cell cycle, high glucose levels promoted the premature differentiation of NSCs. The numbers of MAP2 (a neuronal marker)-positive cells were significantly increased in high glucose-treated differentiated NSCs (Fig. 3e). The levels of the Map2 mRNAs (Fig. 3d) and the TUJ1 (another neuronal marker) proteins (Fig. 3f) were significantly increased in high glucose-treated differentiated NSCs.

### High glucose alters the transcription of proneural and neuronal bHLH factors in differentiated NSCs

**Fig. 4:**
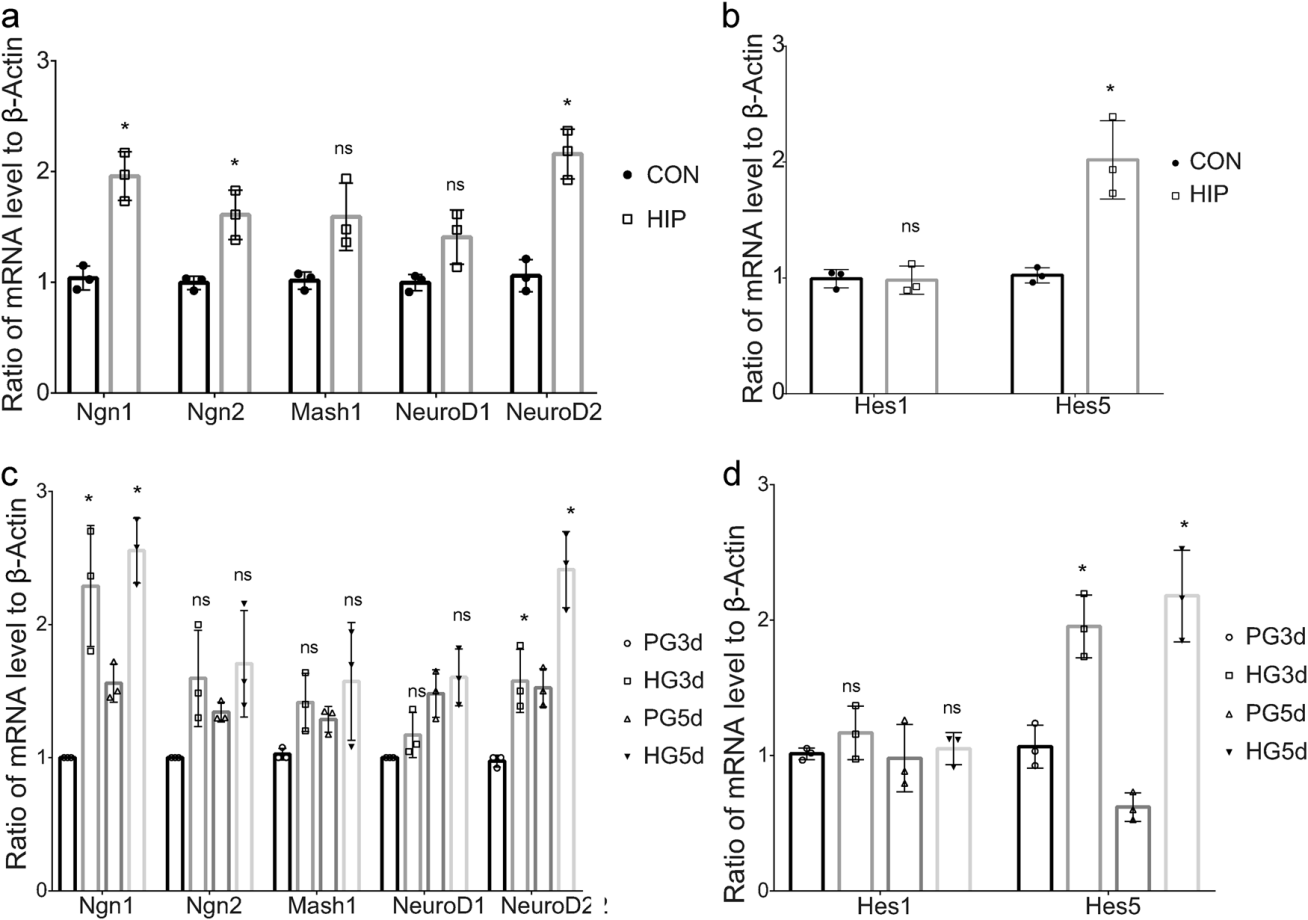
Effects of high glucose levels on the transcription of bHLH factors during NSC differentiation. **a** qPCR analysis of the levels of the activator bHLH factors mRNAs in the E13.5 cerebral cortex of HIP mice compared with the control group. **b** qPCR analysis of the levels of the Hes genes mRNAs in the E13.5 cerebral cortex of HIP mice compared with the control group. **c** qPCR analysis of the levels of the activator bHLH factors mRNAs in high glucose-treated differentiated NSCs after 3 and 5 days of differentiation. **d** qPCR analysis of the levels of the Hes genes mRNAs in high glucose-treated differentiated NSCs. Data represent the mean of three independent experiments ± SD. *p < 0.05 vs. control or PG; ns, no significant difference vs. control or PG

When NSCs decide to become neurons, the neuronal differentiation of NSCs is accompanied by elevated expression of activator bHLH factors (e.g., proneural bHLH factors known as Ngn genes, and neuronal bHLH factors known as NeuroD genes) and decreased repressor bHLH factors (e.g., Hes genes)^49^. Therefore, we examined the effect of moderately high glucose levels on the transcription of these bHLH factors in the cerebral cortex and differentiated NSCs, respectively. As expected, the transcription of Ngn1 and NeuroD2 was significantly increased in the cerebral cortex of HIP mice (Fig. 4a). The transcription of Ngn1, Ngn2, and NeuroD2 was significantly increased in the high glucose-treated differentiated NSCs (Fig. 4c). The transcription of Hes1 was not significantly altered, but the transcription of Hes5 was significantly increased in the cerebral cortex of HIP mice (Fig. 4b) and high glucose-treated differentiated NSCs (Fig. 4d). Based on the pivotal roles of proneural and neuronal bHLH factors in neurogenetic differentiation, the high glucose-induced increase in the transcription of Ngn1 and NeuroD2 might be crucial for determining the fate of NSCs.

### High glucose increases the level of lysine 14 acetylation in histone H3 and the transcription of proneural and neuronal bHLH genes

**Fig. 5:**
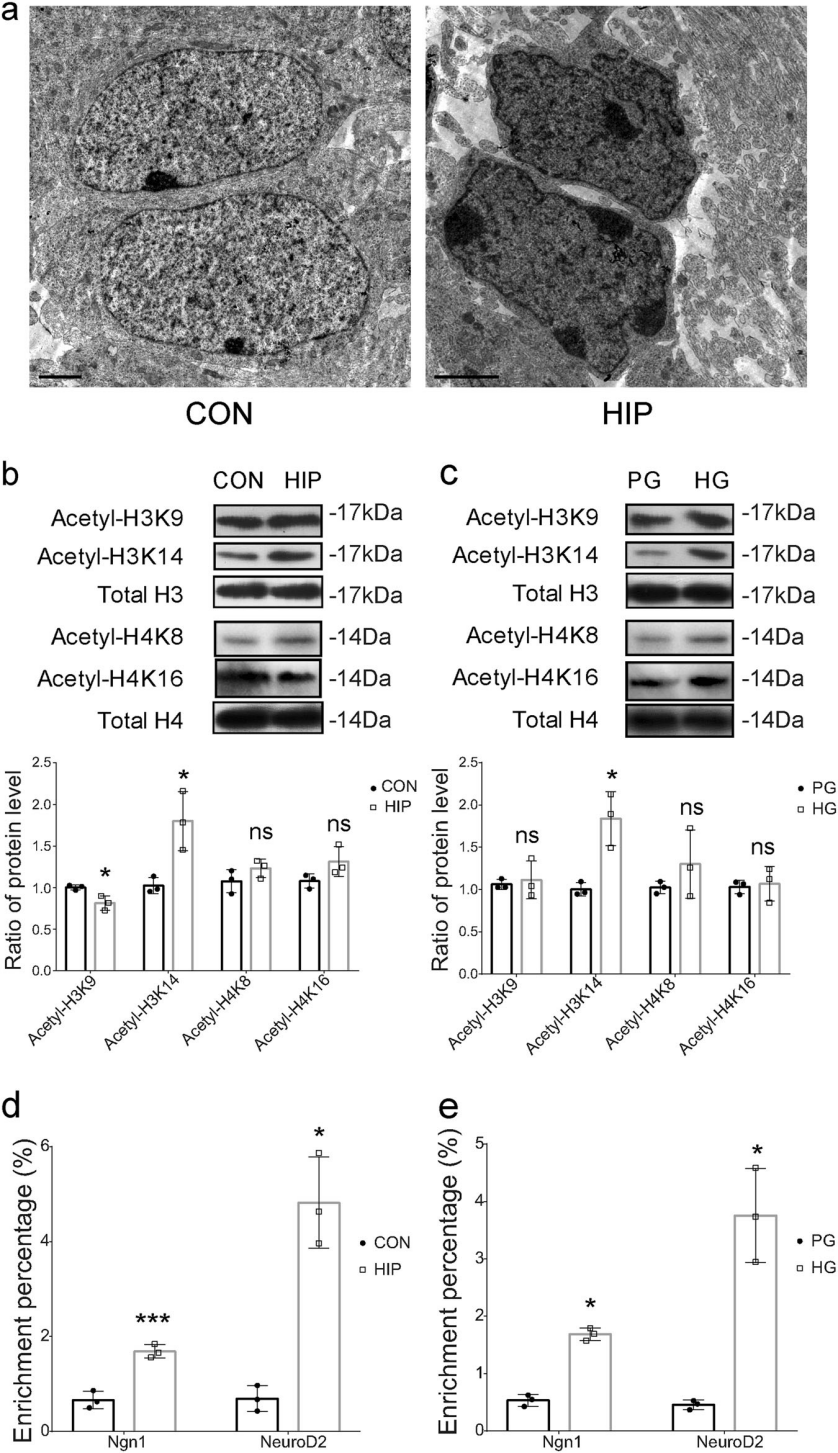
The epigenetic changes induced by high glucose levels during NSC differentiation. **a** Analysis of the chromatin status using transmission electron microscopy. Scale bars, 2 μm. **b**, **c** Western blot analysis of H3K9ac, H3K14ac, total H3, H4K8ac, H4K16ac, and total H4 levels in the E13.5 telencephalon and NSCs cultured in differentiation medium with PG or HG for 3 days. Densitometry analysis comparing levels of the H3K9ac and H3K14ac protein with the total histone H3 protein, and the H4K8ac and H4K16ac protein with the total histone H4 protein. **d**, **e** ChIP analysis of the enrichment efficiency of H3K14ac at the Ngn1 and NeuroD2 promoters in the E13.5 telencephalon and NSCs cultured in differentiation medium with PG or HG. The results were normalized to the corresponding input sample. Data represent the mean of three independent experiments ± SD. *p < 0.05 vs. control; ***p < 0.001 vs. control; ns, no significant difference vs. control

We observed increased numbers of heterochromatin clumps at the nuclear periphery in HIP group under the transmission electron microscope (Fig. 5a), suggesting that hyperglycemia triggers chromatin remodeling in the E13.5 dorsal telencephalon. Recently, epigenetic modifications, particularly histone acetylation, have been reported to participate in the activation of proneural gene transcription^37^. In addition, high glucose has been shown to increase histone acetylation during neural development. Therefore, we detected changes in level of histone acetylation in telencephalon of HIP and differentiated NSCs treated with high glucose levels. We detected changes in level of histone H3 acetylated at lysine 9 (H3K9ac), histone H3 at lysine 14 (H3K14ac), histone H4 acetylated at lysine 8 (H4K8ac), and histone H4 acetylated at lysine 16 (H4K16ac) (Fig. 5b, c), which had been reported to participate in transcriptional activation^50^. Western blot analyses revealed a significant increase in the level of H3K14ac in the HIP group and high glucose-treated group, and a significant decrease in the level of H3K9ac in the HIP group (Fig. 5b, c). We did not detect differences in the levels of acetylation at other sites that were as significant as the increased acetylation of H3K14. Thus, preferential hyperacetylation of H3K14 compared with other sites occurred in response to the high glucose treatment. H3K14ac is necessary and specific for nucleosome disassembly at the promoter region to facilitate gene transcription^51^. Therefore, we examined whether high glucose levels increased the transcriptional activation of proneural and neuronal genes by regulating the acetylation of H3K14 at their promoters, Ngn1 and NeuroD2. We observed an increased enrichment of H3K14ac at the promoters of the Ngn1 and NeuroD2 genes in the HIP group and high glucose-treated group (Fig. 5d, e).

### High glucose increases the acetylation of lysine 14 in histone H3 by disturbing the balance between HATs and HDACs

**Fig. 6:**
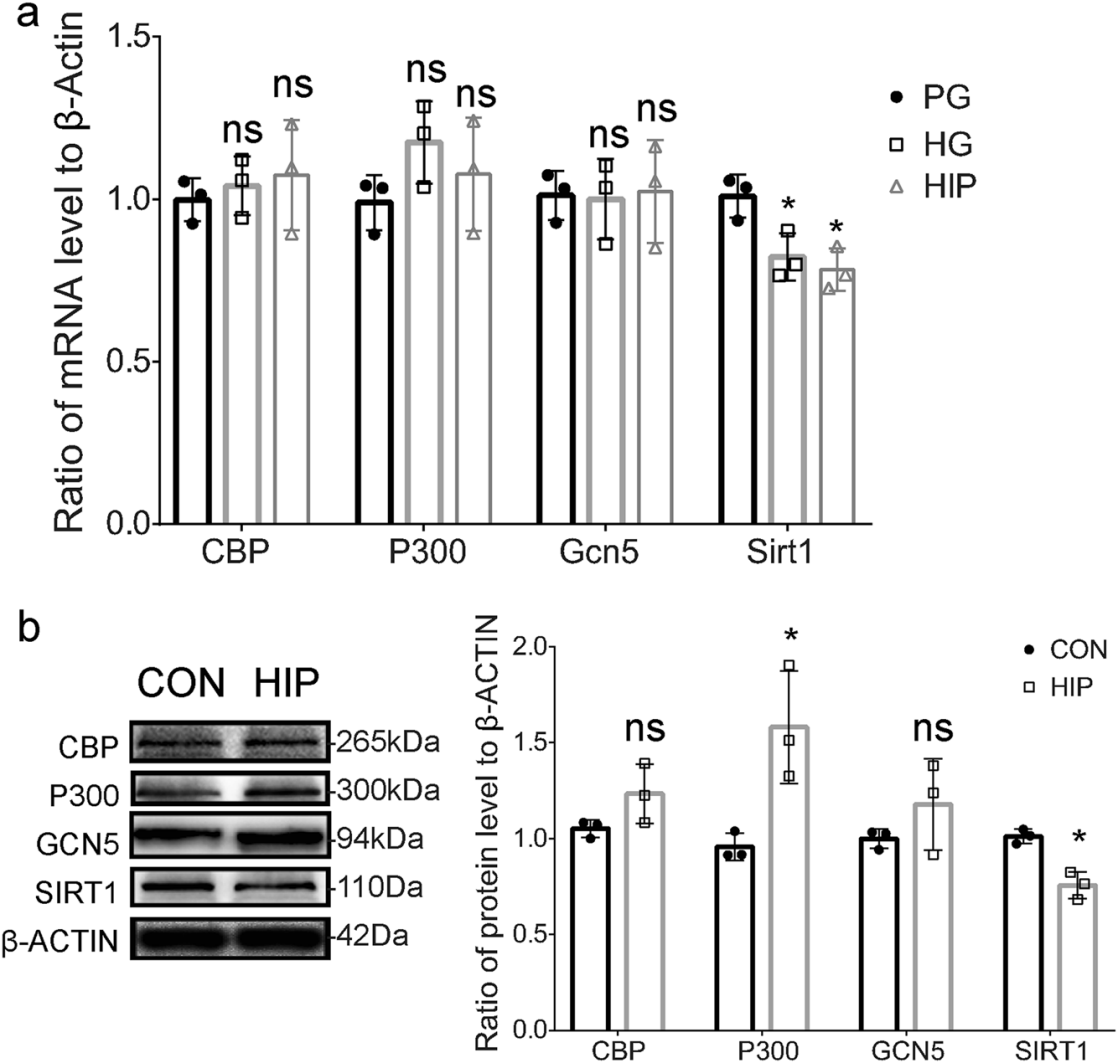
Effects of high glucose concentrations on the expression of the histone acetylases and the histone deacetylases related to H3K14ac. **a** qPCR analysis of levels of HAT and HDAC mRNAs related to H3K14ac in differentiated NSCs treated with PG and HG, and NSCs isolated from HIP mice treated with differentiation medium with PG. **b** Western blot analysis of levels of H3K14ac-related HAT and HDAC proteins in the E13.5 cerebral cortex of HIP mice and the control group. Densitometry analysis of protein levels compared with β-actin. Data represent the mean of three independent experiments ± SD. *p < 0.05 vs. control; ns, no significant difference vs. control

Several HATs and HDACs participate in modifying H3K14ac in the developing nervous system. CBP, P300, and GCN5 are HATs, and SIRT1 is an HDAC^52^. We detected the expression of these enzymes in non-malformed offspring from a moderately hyperglycemic pregnancy and normal pregnancy by quantitative RT-PCR and Western blot analyses. As shown in Fig. 6a, maternal hyperglycemia decreased the mRNA level of the histone deacetylase Sirt1. We also detected expression of these enzymes in differentiated NSCs treated with high glucose levels. As shown in Fig. 6b, maternal hyperglycemia increased the expression of histone acetylase P300, but decreased the expression of the histone deacetylase SIRT1.

## Discussion

Maternal hyperglycemia alters neurodevelopment, but few reports have examined abnormal cerebral development in the offspring without obvious NTDs. We discovered that maternal hyperglycemia disturbed neocortical lamination in non-malformed offspring via facilitating the cell cycle exit of NSCs and altered the fate of early-born neocortical neurons during neocortical neurogenesis. We further demonstrated that increased accumulation of H3K14 acetylation at the Ngn1 and NeuroD2 promoters activated their transcription; then, Ngn1 and NeuroD2 initiated the premature differentiation of NSCs and sustained the commitment to neuronal fates under high glucose condition.

In the present study, we selected the pregnant mice with moderate hyperglycemia in consideration of that the incidence of NTDs in the HIP group was less than the severe diabetes group (with fasting blood glucose levels exceeding 16.7 mmol/l) in our previous experiments^21^, so they produced more offspring exposed to maternal hyperglycemia while had normal phenotype without NTDs compared with pregnant mice with severe hyperglycemia. The data from epidemiological survey demonstrated that offspring affected by maternal hyperglycemia had high risks of long-term neuropsychiatric disorders than the offspring of normal pregnancy^3^, which suggested the effects of maternal hyperglycemia on aberrant neurodevelopment. So it is considerable to further explore the effects of maternal hyperglycemia on embryonic corticogenesis.

The birthdate of cortical projection (pyramid) neurons is closely associated with laminar identity^29^. Alterations in neocortical lamination contribute to neuropsychological disorders. The position where we acquired image of laminar structure is located in the boundary area of frontal cortex and parietal cortex, which is usually used to observe laminar structure^47^. Our results revealed that maternal hyperglycemia altered the fates of deep layer (early-born) neocortical neurons of this position during neocortical neurogenesis, which suggested that maternal hyperglycemia might influence embryonic brain function via disturbing corticogenesis. We further postulated that maternal hyperglycemia affected the birthdates of neocortical projection neurons by altering NSC differentiation, resulting in the abnormal laminar distribution of neocortical neurons in the offspring from a moderately hyperglycemic pregnancy.

During the neocortical development, multipotent NPCs maintain the balance between self-renewal and neurogenesis. In the present study, a high glucose treatment promoted the early exit of NSCs from the cell cycle and significantly accelerated the progression of neurogenesis. A significant increase in the number of early-born neuron was observed, while no significant change in number of late-born neuron was identified in our experiments. Overall, high glucose levels markedly enhanced the premature differentiation of NSCs to produce more early-born deep layer neocortical neurons in an earlier period of neurogenesis. However, more complex and in-depth studies are needed to confirm the effects of maternal hyperglycemia on the late-born neuron fates.

The bHLH family of transcription factors plays a key role in cell fate decisions in NPCs and the maturation of terminally differentiated cells^43^. During the neocortical development, Ngn1 and Ngn2 are transiently expressed and are critical for the commitment of NPCs in the ventricular zone, followed by the expression of NeuroD1 and NeuroD2 during terminal differentiation^44,53^. Ngn1 initiates neuronal commitment-related gene expression, while NeuroD2 further sustains neuronal fates^54,55^. As shown in our previous studies, the expression of bHLH factors is altered in proliferating and differentiated NSCs exposed to high glucose concentrations^19,23^. Maternal hyperglycemia also increases the transcription of bHLH genes in the embryonic telencephalon^21,23^. In the present study, we detected significantly increased the expression of the Ngn1 and NeuroD2 mRNAs in differentiated NSCs under high glucose condition during the formation of the laminar structure of the neocortex in fetuses from the HIP group that did not exhibit significant malformations. Therefore, it is an innovation to study the effects of high glucose levels on neurogenetic differentiation by investigating the transcription of bHLH genes in non-malformed offspring from moderately hyperglycemic pregnant mice.

Transcriptional profiles are altered in embryos exposed to maternal hyperglycemia, implicating epigenetic regulation of gene expression^13^. Moreover, epigenetic regulation plays an important role in the development of the nervous system^33,34^. According to numerous studies, the modifications of histones that affect the structure and density of chromatin dynamically regulate gene expression associated with the cell fate decisions in NSCs^38^. Among the many modifications, histone acetylation relaxes the chromatin architecture and allows transcriptional factors to easily access the genomic DNA; acetylation mainly occurs on histone H3 and histone H4. Numerous studies on NTDs induced by a hyperglycemic pregnancy have identified variations in epigenetic modifications, particularly changes in histone acetylation. Lower acetylation of lysine 9 on H3 (H3K9) was observed in proliferating NSCs exposed to excessively high glucose concentrations (40 mmol/l) and in NSCs isolated from embryos from a diabetic pregnancy^38^. However, no report has examined the effect of moderate maternal hyperglycemia on histone acetylation in determining the fate of NSCs during neocortical neurogenesis. Interestingly, moderate maternal hyperglycemia and a high glucose (25 mmol/l) treatment both preferentially increased the acetylation of H3K14, but had no significant influence on the acetylation of H3K9, H4K8, or H4K16, which had been shown to participate in transcriptional activation^50^. Moreover, high glucose levels increased the acetylation of H3K14 at the Ngn1 and NeuroD2 promoters, which then predictably activated their transcription. These findings are the first to directly show that high glucose levels alter the expression of pivotal genes participating in neurogenetic differentiation by affecting histone acetylation.

Histone acetylation is reversible and is regulated by two groups of enzymes, HATs and HDACs. P300 and SIRT1 play important roles in the differentiation and fate determination of NSCs during brain development^56–58^. Dheen et al. observed significantly decreased levels of the SIRT1 mRNA and protein in NSCs from embryos from pregnant diabetic mice^59^. Moreover, SIRT1 represses the activity of P300 by deacetylation to maintain the balance between histone acetylation and deacetylation during cellular differentiation^60^. Meanwhile, the imbalance between histone acetylation (upregulated P300) and deacetylation (downregulated SIRT1) under hyperglycemic conditions increases the expression of genes activated by H3K14 acetylation^61^. The results from the present study improve our understanding of the balance between P300- and SIRT1-based regulation of NSC differentiation.

In conclusion, our data suggested the association between the effects of maternal hyperglycemia on neocortex development and epigenetic regulation on neurogenesis. High glucose levels promoted the early exit of NSCs from the cell cycle and the early-born neuron fate commitment. High glucose levels increased H3K14 acetylation levels at the Ngn1 and NeuroD2 promoters by disturbing the balance between P300 and SIRT1, and promoted transcription of Ngn1 and NeuroD2 (Fig. S2a). Enhanced and premature expression of Ngn1 and NeuroD2 eventually led to the premature neurogenetic differentiation of NSCs and advance birth of newborn neurons in the neocortex, especially increase of number of projection neuron in layer 5 (Fig. S2b). The epigenetic modifications are not only essential for the cell fate decisions in NSCs but are also dynamically adjustable in response to environmental stimuli, such as maternal hyperglycemia. The present study contributes to improving our understanding of the adverse effects of maternal hyperglycemia on neocortical neurogenesis. Based on our findings, high glucose levels regulate the histone acetylation-dependent gene expression network, which probably is promising target for intervention in fetal neurodevelopment deficits.

## Materials and methods

### Ethics statement

We abide by the Guide for the care and use of laboratory animals, Eighth edition (2011) in animal care and treatment. Our experimental protocol was authorized by the Ethics Committee on Animal Experiments of the Medical School of Shandong University (No. KYLL-2017(KS)-357).

### Animals

Hyperglycemia was induced in 8-week-old female C57BL/6J mice (Experimental Animal Center of Shandong University, Jinan, China) by an intraperitoneal (ip) injection of streptozotocin (STZ; 75 mg/kg body weight; Sigma-Aldrich, St. Louis, MO, USA) dissolved in citrate buffer (0.01 mol/l, pH 4.5) for three consecutive days. Fasting blood glucose (FBG) levels were examined 7 days after the STZ injection using an FAD-GDH System (Sanocare, Changsha, China) and were monitored every 3 days and immediately before euthanasia. Mice with FBG levels ranging from 8.3 mmol/l (150 mg/dl) to 11.1 mmol/l (200 mg/dl) were regarded as the HIP group. Control group mice were injected with an equal volume of citrate buffer, and their FBG levels were <6.7 mmol/l (120 mg/dl)^23^. Pregnancy was designated as E0.5 when a copulation plug was observed after mating. At designated times (E11.5, E13.5, and E17.5), embryos were collected from pregnant mice by caesarean section. The forebrains of embryos and neonates were fixed with 4% paraformaldehyde (PFA) in PBS overnight at 4 °C and cryoprotected with 20% sucrose in PBS. Serial sections of the brain were cut at a thickness of 20 μm using a cryostat (Leica Microsystems Nussloch GmbH, Nussloch, Germany).

### Analysis of morphology

The implantations and alive fetuses were counted at E11.5, and the percentage of alive fetuses among implantations was calculated. NTDs were screened in alive fetuses with a stereomicroscope (Olympus, Tokyo, Japan) in a blinded manner.

### EdU/Ki67 immunolabelling

At E13.5, pregnant mice were ip injected with EdU (100 mg/kg body weight; Sigma-Aldrich), and embryos were collected 24 h later. Frozen sections of the embryonic forebrain were incubated with HCl (2N), blocked with 10% goat serum and incubated with a mouse anti-EdU monoclonal antibody (1:1000; Sigma-Aldrich) and a rabbit anti-Ki67 monoclonal antibody (1:1000; Cell Signaling Technology, Beverly, MA, USA). Sections were then incubated with FITC-conjugated goat anti-mouse IgG and TRITC-conjugated goat anti-rabbit IgG (1:200; Millipore, Billerica, MA, USA), and counterstained with DAPI (5 μg/ml; Vector Laboratories, Burlingame, CA, USA); sections were mounted with fluorescent mounting medium (Beyotime Institute of Biotechnology, Shanghai, China). Finally, images of these sections were captured using a fluorescence microscope (DP72, Olympus). For the analysis of proliferating (cell cycle re-entry) and quiescent (cell cycle exit) cells, the percentage of EdU+/Ki67+ and EdU+/Ki67– cells among the total number of EdU+ cells in the dorsal telencephalon were counted and was calculated^62^.

### TUNEL assay

DNA fragmentation was detected using TUNEL staining (Promega, Madison, WI, USA). Briefly, frozen sections from E13.5 embryos were incubated with the TUNEL reaction mixture and then counterstained with DAPI. Images were captured with a fluorescence microscope (DP72, Olympus). The numbers of TUNEL-positive and DAPI-positive cells in the dorsal telencephalon were counted and percentages were calculated.

### Primary NSCs culture

NSCs were isolated from the telencephalon of C57BL/6J mice at embryonic day 12.5. Cells were separated mechanically in DMEM/F12 (1:1) (Gibco, Gaithersburg, MD, USA). After centrifugation and resuspension, cells were counted. Cell were seeded at final density of 2 × 10^5^ cells/ml in DMEM/F12 (1:1) supplemented with 2% B27 (Gibco, Gaithersburg, MD, USA), 20 ng/ml basic fibroblast growth factor (bFGF; R&D Systems, Minneapolis, MN, USA), 20 ng/ml EGF (Invitrogen, Carlsbad, CA, USA), 100 U/ml penicillin, and 100 μg/ml streptomycin. Cells were seeded into 75 cm^2^ T-flasks and incubated in a humidified atmosphere containing 5% CO_2_ and 95% air at 37 °C. After 3 days of incubation, primary neurospheres were dissociated into single cells and cultured for 3 days to form passage 1 neurospheres as shown in Fig. S3. These neurospheres were dissociated again and transferred to poly-L-ornithine (PLL)-coated 6-well plates and 24-well plates with PLL-coated coverslips at the bottom of each well. After 24 h, the medium containing growth factors was replaced with differentiation medium containing 2% FBS. NSCs were divided into two groups and cultured further to examine the effects of high D-glucose on differentiated NSCs. The two groups were cultured in medium containing a normal physiological glucose concentration (PG) of 5 mmol/l D-glucose or in high glucose (HG) medium containing 25 mmol/l D-glucose (Sigma-Aldrich, St Louis, MO, USA), respectively, for 3, 5, and 7 days.

We also isolated NSCs from the telencephalon of non-malformed embryos of HIP and cultured them in differentiation medium containing a physiological glucose concentration to examine epigenetic reprogramming in utero caused by maternal hyperglycemia.

### Immunofluorescence staining

Frozen sections of the telencephalon from E17.5 embryos or P1 neonates were permeabilized with 0.1% TritonX-100, blocked with 10% goat serum and incubated with the following primary antibodies: rabbit TBR1 monoclonal antibody (1:1000; Abcam, Cambridge, UK), rat CTIP2 monoclonal antibody (1:1000; Abcam, Cambridge, UK), and rabbit SATB2 polyclonal antibody (1:1000; Abcam, Cambridge, UK). FITC-conjugated goat anti-rabbit IgG and tetramethylrhodamine isothiocyanate (TRITC)-conjugated goat anti-rat IgG were used as secondary antibodies. Cell nuclei were counterstained with DAPI, and sections were mounted with fluorescent mounting medium (Beyotime Institute of Biotechnology, Shanghai, China). Images were captured using a fluorescence microscope (DP72, Olympus). Cells on 300 µm wide sections of the mediolateral axis of cortical sections expressing indicated markers were counted. We analyzed the density of cells by calculating the number of cells per 100 µm mediolateral axis wide^63^.

Cells on the coverslips were fixed with 4% PFA. The cell slides were handled as described above. We used the following primary antibodies for staining: anti-MAP2 antibody (1:500; Millipore, Billerica, MA, USA) and anti-phosphohistone H3 antibody (1:1000, Cell Signaling Technology, Beverly, MA, USA). Images were captured with a fluorescence microscope (DP72, Olympus).

### RNA extraction and quantitative real-time reverse transcriptase polymerase chain reaction (RT-PCR)

Total RNA was extracted from dorsal telencephalon tissues of E13.5 embryos and cultured differentiated NSCs using Trizol reagent (Invitrogen, Carlsbad, CA, USA) and reverse transcriptions were primed with oligo dT and performed on equal amounts of total RNA (2 μg) using ReverAid First Strand cDNA Synthesis Kit (Fermentas, Burlington, Ontario, Canada). Real-time PCR was performed with SYBR Green Realtime PCR Master Mix (TOYOBO CO., Ltd., Japan) and performed with CFX connect (Bio-Rad, Hercules, CA, USA). Primer sequences are listed in Table S1. The fold changes of mRNA levels were calculated with the 2^−ΔΔCt^ method^25^, with β-actin serving as the normalization control.

### Protein extraction and western blot analysis

Mouse dorsal telecephalon tissues from E13.5 embryos and cultured NSCs were lysed in ice-cold RIPA buffer (50 mM pH 7.4 Tris–HCl, 150 mM NaCl, 0.1% SDS, 5 mM EDTA, 2 mM PMSF, 20 mg/ml aprotinin, 20 mg/ml leupeptin, 10 mg/ml pepstatin A, 150 mM benzamidine, and 1% NP-40) for 20 min at 4 °C. Supernatants were collected and total protein concentrations were measured by the BCA method. Equivalent amounts of protein in each sample were separated on 8–15% SDS–PAGE gel electrophoresis and then electrotransferred onto 0.22 or 0.45 μm PVDF membranes (Millipore, Billerica, MA, USA). The membranes were then blocked with 5% milk in TBST buffer and probed with the following primary antibodies at 4 °C overnight: anti-TUJ1 antibody (1:500, Beyotime Institute of Biotechnology, Shanghai, China), anti-histone H3 antibody (1:2000, Cell Signaling Technology, Beverly, MA, USA), anti-acetyl-histone H3 (lys9) antibody (1:1000, Cell Signaling Technology, Beverly, MA, USA), anti-acetyl-histone H3 (lys14) antibody (1:1000, Cell Signaling Technology, Beverly, MA, USA), anti-histone H4 antibody (1:1000, Cell Signaling Technology, Beverly, MA, USA), anti-acetylhistone H4 (lys8) antibody (1:1000, Cell Signaling Technology, Beverly, MA, USA), anti-P300 antibody (1:1000, Santa Cruz, CA, USA), anti-CBP antibody (1:500, Cell Signaling Technology, Beverly, MA, USA), anti-SIRT1 (1:1000, Cell Signaling Technology, Beverly, MA, USA), anti-GCN5 (1:1000, Cell Signaling Technology, Beverly, MA, USA), and anti-β-actin antibody (1:2000, Sigma-Aldrich, St Louis, MO, USA). Secondary antibodies were HRP conjugated to either goat anti-mouse IgG or anti-rabbit IgG. The bands were finally visualized by the enhanced chemiluminescence ECL detection kit (Merch Millipore, Billerica, MA, USA). The intensity of bands was determined by the Image-Pro Plus 6.0 software.

### Electron microscopy

Dorsal telecephalon tissues from E13.5 embryos were cutted into 1 mm^3^ and fixed with 3% glutaraldehyde for 2 h at 4 °C. The fixed tissues were washed no less than three times with PBS. Then, the tissues were post-fixed in 1% OsO_4_ for 1 h at 4 °C. The tissues were dehydrated through an ascending ethanol series, and infiltrated with 100% epoxy resin:acetone (1:3, 1:1, 3:1) 1 h, 4 h, no less than 12 h, respectively. Finally, the tissues were embedded in fresh epoxy resin and polymerized at 37 °C for 12 h, 45 °C for 12 h, 60 °C for 1–2 h. Ultrathin sections were supported on grids (150 nm) and stained with uranyl acetate for 20 min and lead citrate for 20 min at room temperature. Images were captured with JEM-1200EX transmission electron microscope (Japan Electron Optics Laboratory Company, Tokyo, Japan).

### Chromatin immunoprecipitation (ChIP) assay

ChIP was performed with an EZ-ChIP kit (Merck Millipore, Billerica, MA, USA) according to the manufacturer’s instructions. Mouse dorsal telecephalon from E13.5 embryos were fixed with 1% formaldehyde to cross-link histones to the DNA for 10 min at room temperature and then harvested. Each pellet was resuspended in SDS Lysis Buffer containing protease inhibitors. Cells were lysed and sonicated to shear the chromatin into fragments with an average size of ~150–900 bp. The supernatant was immunoprecipitated with an anti-acetyl-histone H3 (lys14) antibody (Cell Signaling Technology, Beverly, MA, USA) or a control rabbit anti-IgG antibody overnight at 4 °C. After washes, elution, and reversing the cross-links, the DNA was purified from each sample and the corresponding input sample and analyzed using qRT-PCR. The primers used for PCR^34,64^ are listed in Table S1.

### Statistical analysis

The quantitative data are reported as absolute values, rates, mean ± SD, unless specified otherwise. The percentages of alive fetuses and NTDs were analyzed using the *Χ*^2^ test. Other variables were analyzed using Student’s *t* test or one-way analysis of variance followed by Tukey’s test. Data are reported as the mean ± SD, and differences were considered statistically significant when *p* values were <0.05.

## Supplementary information

Figure S1

Figure S2

Figure S3

table S1

Supplemental figure legends
