# Supplementary figures and images for: Maternal hyperglycemia disturbs neocortical neurogenesis via epigenetic regulation in C57BL/6J mice
Source: Cell Death Dis. 2019 Mar 1;10(3):211. doi: 10.1038/s41419-019-1438-z (PMC6397163; doi:10.1038/s41419-019-1438-z)

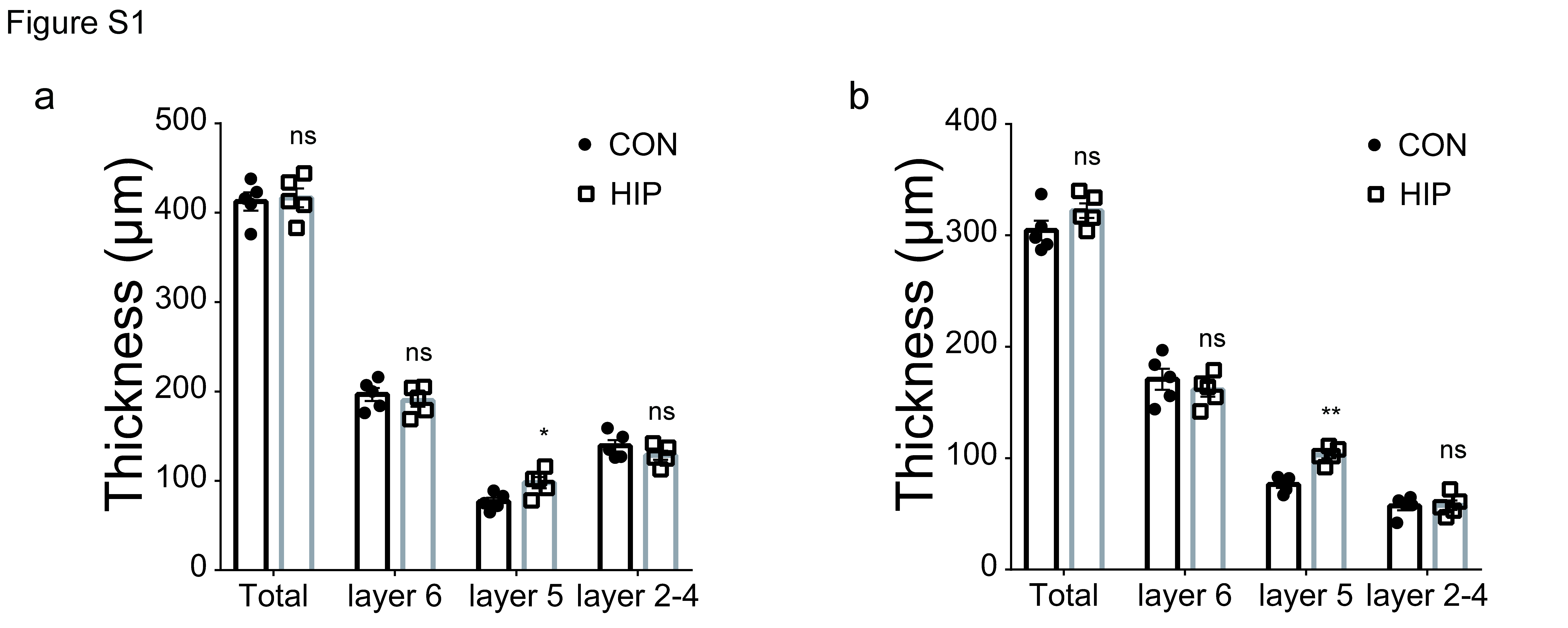

Supplement: Supplementary file 1 — Figure S1 [file 41419_2019_1438_MOESM1_ESM.tif]

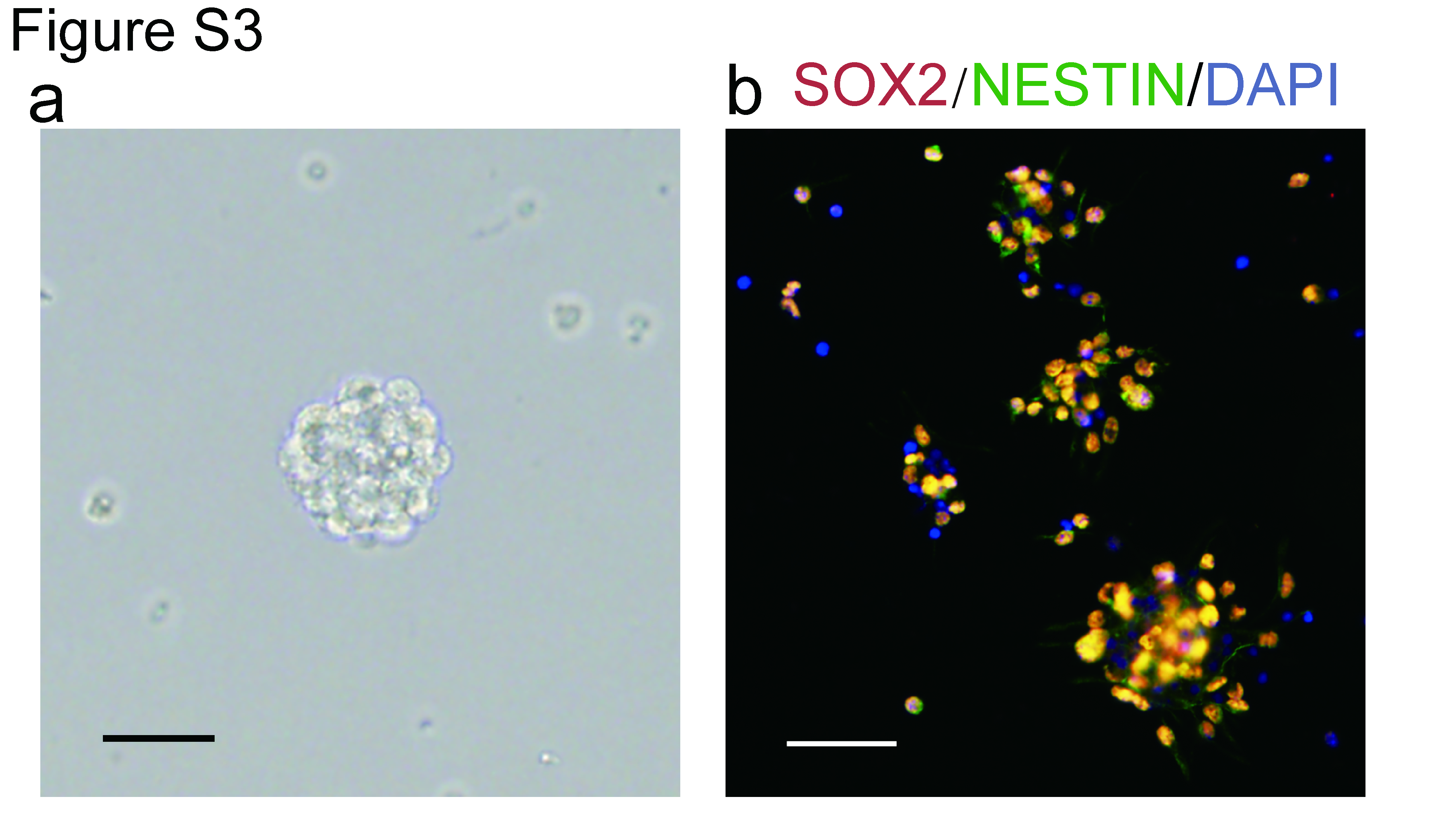

Supplement: Supplementary file 3 — Figure S3 [file 41419_2019_1438_MOESM3_ESM.tif]
