# Supplementary material for: Maternal hyperglycemia disturbs neocortical neurogenesis via epigenetic regulation in C57BL/6J mice
Source: Cell Death Dis. 2019 Mar 1;10(3):211. doi: 10.1038/s41419-019-1438-z (PMC6397163; doi:10.1038/s41419-019-1438-z)
Supplement: Supplementary file 4 — table S1 [file 41419_2019_1438_MOESM4_ESM.docx]

Table S1

Primers used in quantitative real-time PCR analysis

| Gene | Forward primer | Reverse primer |
| --- | --- | --- |
| β-Actin | 5’-ACCTTCTACAATGAGCTGCG-3’ | 5’-CTGGATGGCTACGTACATGG-3’ |
| p21 | 5’-GAGAACGGTGGAACTTTGACTTC-3’ | 5’-GAGGAAGTACTGGGCCTCTTG-3’ |
| p57 | 5’-GGAGCAGGACGAGAATCAAG-3’ | 5’-GTTCTCCTGCGCAGTTCTCT-3’ |
| MAP2 | 5’-GAATAAGCAAGAGCCCAGAG-3’ | 5’-GTCCGTCGTGCTGAAGAG-3’ |
| Ngn1 | 5’-ATCCCCTTTTCTCCTTTCCTG-3’ | 5’-CCTAGTGGTATGGGATGAAACAG-3’ |
| Ngn2 | 5’-GCTGTGGGAATTTCACCTGT -3’ | 5’- AAATTTCCACGCTTGCATTC-3’ |
| NeuroD1 | 5’-TGCTACTCCAAGACCCAG-3’ | 5’-AAGAAAGTCCGAGGGTTGA-3’ |
| NeuroD2 | 5’-GGGAACAATGAAATAAGCGAGAAG-3’ | 5’-CAGCATGGTGCCTCAGAG-3’ |
| Hes1 | 5’-AAGCCTATCATGGAGAAGAGG-3’ | 5’-GTTGATCTGGGTCATGCAGT-3’ |
| Hes5 | 5’-GATGCTCAGTCCCAAGGAGA-3’ | 5’-CGTGGAAGTGGTAAAGCAG-3’ |
| Gcn5 | 5’-TCCATTTTCACTGTCACCCG-3’ | 5’-ATTTGCCCCGTAGATCTCTTC-3’ |
| CBP | 5’-GACCGCTTTGTTTATACCTGC-3’ | 5’-TCTTATGGGTGTGGCTCTTTG-3’ |
| P300 | 5’-GTTGCTATGGGAAACAGTTATGC-3’ | 5’-TGTAGTTTGAGGTTGGGAAGG-3’ |
| Sirt1 | 5’-CTCTGAAAGTGAGACCAGTAGC-3’ | 5’-TGTAGATGAGGCAAAGGTTCC-3’ |
| Ngn1  (ChIP) | 5’-CATTGTTGCGCGCCGTA-3’ | 5’-GCGATCAGATCAGCTCCT-3’ |
| NeuroD2(ChIP) | 5’-GGTGCCAGCATCTACCTATG-3’ | 5’-CATTTCCCTGTCTCCAGGTC-3’ |
