# Supplementary material for: Maternal hyperglycemia disturbs neocortical neurogenesis via epigenetic regulation in C57BL/6J mice
Source: Cell Death Dis. 2019 Mar 1;10(3):211. doi: 10.1038/s41419-019-1438-z (PMC6397163; doi:10.1038/s41419-019-1438-z)
Supplement: Supplementary file 5 — Supplemental figure legends [file 41419_2019_1438_MOESM5_ESM.docx]

Fig. S1. Effects of maternal hyperglycemia on the thickness of the neocortical layers.

**a** The total thickness of the neocortex (layers 2–6), thickness of deeper layers (layer 6 and layer 5, respectively) and superficial layers (layers 2–4) in the cerebral cortex of neonatal (P0) HIP mice compared with the control group (p=0.8310, p=0.6599, p=0.0149 and p=0.4024, respectively). **b** The total thickness of the neocortex (layers 2–6), thickness of deeper layers (layer 6 and layer 5, respectively) and superficial layers (layers 2–4) in the cerebral cortex of E17.5 HIP mice compared with the control group (p=0.2504, p=0.4065, p=0.0096 and p=0.8133, respectively). Data represent the mean of five independent experiments ± SD. *, P < 0.05 versus control; **, P < 0.01 versus control; ns, no significant difference versus control.

Fig. S2. Schematic showing the epigenetic regulation of the transcription of proneural genes in the hyperglycemic environment and its influence on NSC differentiation and neocortical neuron fate.

**a** High glucose concentrations increased the levels of acetyl-H3K14 at the promoters of proneural and neuronal bHLH genes and subsequently activated their transcription. **b** The accelerated transcriptional waves further promoted the premature differentiation of NSCs and increased the number of neurons displaying the early-born deep layer fate.

Fig. S3.Primary cultured NSCs in proliferation medium.

**a** Morphological appearance of phase contrast images of NSCs cultured in proliferation medium. **b** Immunofluorescence images showing the expression of Nestin/Sox2 double-labeled cells cultured in proliferation medium. Scale bars 50 m.
